# Supplementary material for: Parallel loss of type VI secretion systems in two multi-drug-resistant Escherichia coli lineages
Source: Microb Genom. 2023 Nov 16;9(11):001133. doi: 10.1099/mgen.0.001133 (PMC10711316; doi:10.1099/mgen.0.001133)
Supplement: Supplementary material 1 [file mgen-9-1133-s001.pdf]

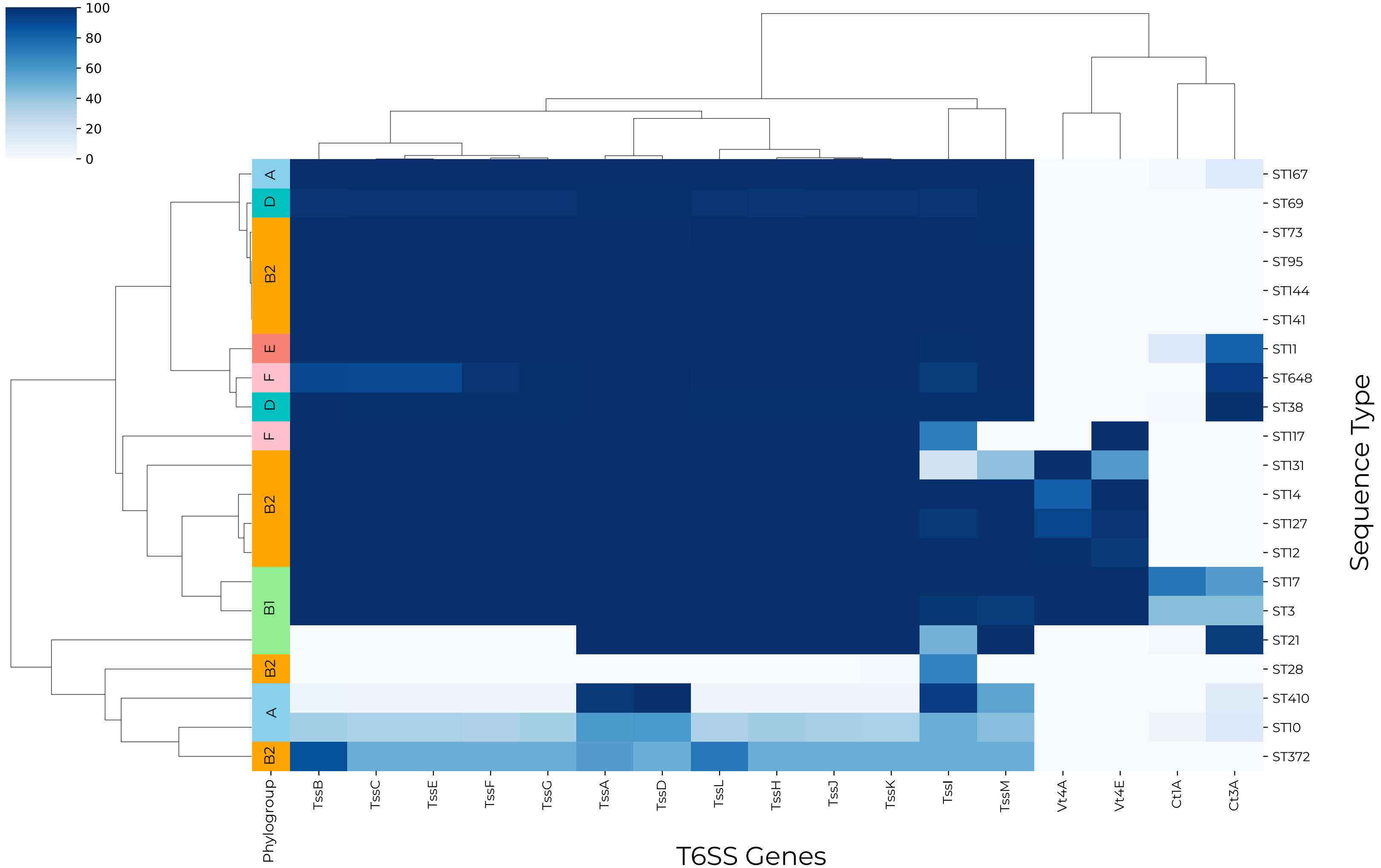

S 1. Hierarchically clustered heatmap displaying the average percentage presence of 17 T6SS structural genes across 21 STs. SecRet6 identification codes: Vt4A: TssI, Vt4E: TssM, Ct1A: TssI, Ct3A: TssI.

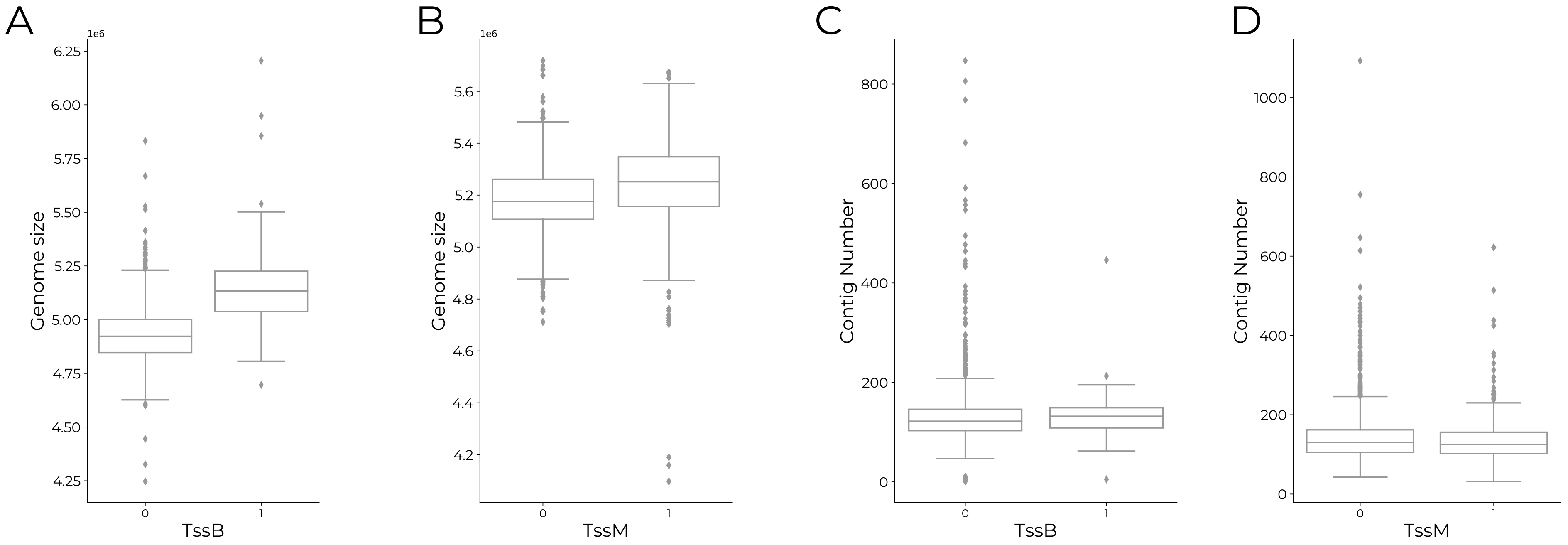

S 2. Boxplots displaying distributions of genome size for assemblies with and without a complete *tss* region for (A) ST410 and (B) ST131. Distributions of number of contigs for assemblies with and without a complete *tss* region for (C) ST410 and (D) ST131. TssB and TssM (Vt4E) are chosen representative determinants of a *tss* region for ST410 and ST131 respectively, based on the results of Figure 2.

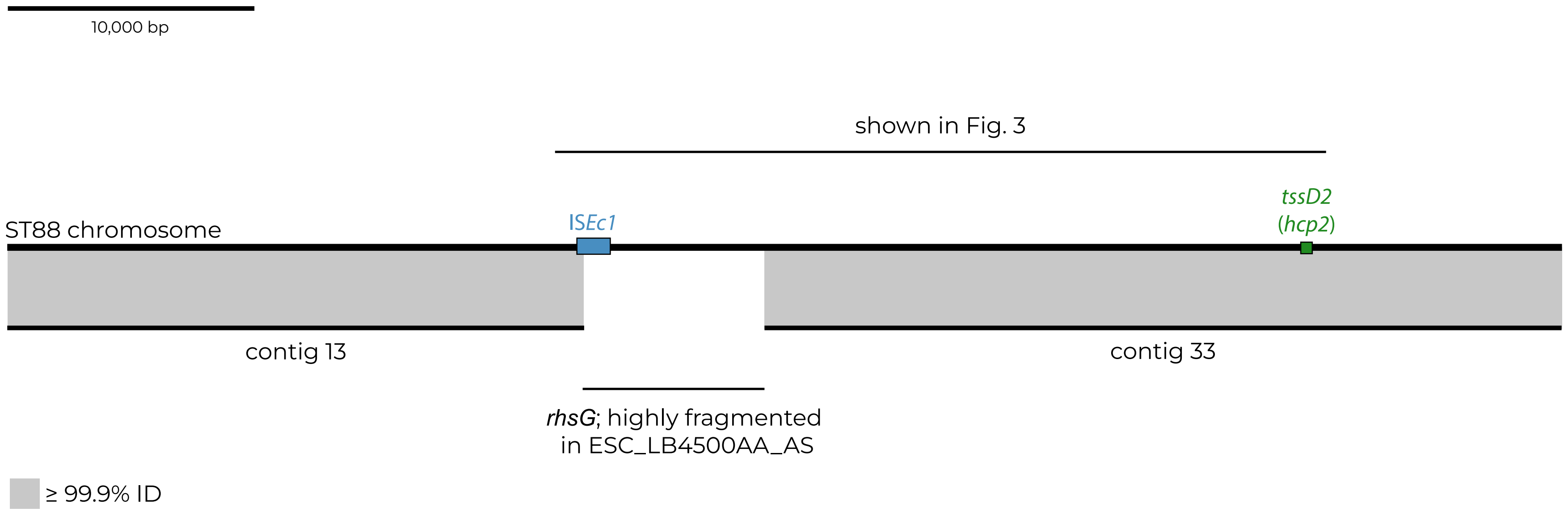

S 3. Scaled schematic showing the alignment of tss region sequences in the draft ST410 genome (EnteroBase assembly barcode LB4500AA_AS and the complete ST88 genome (GenBank accession CP056618) that was used as a scaffold when determining the structure of the region in ST410 clade A. Horizontal lines represent sequences, connected by grey shading indicative of alignments with $\geq$ 99.9% nucleotide identity. Contig numbers in LB4500AA_AS are indicated below, and the highly fragmented 'recombination hot spot' region is labelled. The boundaries of the tss region are marked with IS*Ec1* on the left and *tssD2* on the right, and the extent of the sequence shown in Figure 3 is indicated above.
